# First-in-human experience with GRPR antagonist [^68^Ga]Ga-NOTA-PEG_2_-RM26 in prostate and breast cancer patients using PET/CT

**DOI:** 10.1186/s13550-025-01204-y

**Published:** 2025-02-20

**Authors:** Annie Bjäreback, Olof Jonmarker, Antonios Tzortzakakis, Emma Jussing, Chunde Li, Renske Altena, Cecilia Hindorf, Anna Orlova, Rimma Axelsson, Thuy A. Tran

**Affiliations:** 1 Department of Nuclear Medicine and Medical Physics, Section Nuclear Medicine Huddinge, Karolinska University Hospital, Stockholm, Sweden; 2 Department of Molecular Medicine and Surgery, Karolinska Institutet, Stockholm, Sweden; 3 Division of Radiology, Department for Clinical Science, Intervention and Technology (CLINTEC), Karolinska Institutet, Stockholm, Sweden; 4 Department of Oncology-Pathology, Karolinska Institutet, Stockholm, Sweden; 5 Department of Nuclear Medicine and Medical Physics, Theranostics Trial Center, Karolinska University Hospital, Stockholm, Sweden; 6 Department of Oncology, Södersjukhuset, Stockholm, Sweden; 7 Department of Clinical Science and Education, Karolinska Institutet, Södersjukhuset, Stockholm, Sweden; 8 Department of Medicinal Chemistry, Uppsala University, Uppsala, Sweden; 9 Science for Life Laboratory, Uppsala University, Uppsala, Sweden

## Introduction

Gastrin-releasing peptide receptors (GRPR) are overexpressed in many tumors including prostate, breast, and colorectal cancers [1]. In particular, GRPR are overexpressed in low-grade prostate cancer (PC) and estrogen-positive (ER+) breast cancer (BC) [2, 3]. Positron emission tomography (PET) imaging of GRPR allows in vivo assessment of GRPR expression. Compared to agonists, GRPR antagonists have favourable pharmacokinetics, limiting gastrointestinal side effects and high selection of binding to target cell receptors [4]. Several GRPR antagonists have been developed and tested thoroughly using different radionuclides for PET and Single Photon Emission Computed Tomography [5]. [^68^Ga]Ga-RM2 and [^68^Ga]Ga-NeoBOMB1 are two of the most well-studied GRPR antagonists labelled to gallium-68 [6]. GRPR antagonists based on the peptide d-Phe-Gln-Trp-Ala-Val-Gly-His-Sta-Leu-NH_2_, hereafter referred to as RM26, have shown high affinity towards GRPR and favourable biodistribution with low absorbed doses to healthy organs [7]. Radionuclides are bound using macrocyclic chelators such as NOTA, NODAGA, DOTA, and DOTAGA. In this setting, ^68^Ga-labelled RM26 NOTA has shown favourable characteristics [7]. A molecular spacer sequence prevents interference with the binding site [8]. Coupling the radiometal-chelator complex using two to six polyethylene glycol (PEG) spacer lengths affects hydrophilicity, slightly shifting the excretion route from hepatic to renal. However, the conjugate with the shortest PEG linker, NOTA-PEG_2_-RM26, demonstrated the best association rate, k_a_, and the best half-maximal inhibitory concentration (IC50), translating into higher activity uptake in GRPR-expressing tissues. Still, variations were minor, with all four conjugates tested, as demonstrated by Varasteh et al. [9]. Two RM26 tracers, namely [^68^Ga]Ga-NOTA-PEG_3_-RM26 and [^99m^Tc]Tc-maSSS-PEG_2_-RM26, have already been tested in humans and proven safe [10, 11].

We hypothesised that conjugates with shorter linkers could have slightly different distribution profiles due to smaller size, different lipophilicity, and better tissue penetration. Small changes in the composition and assembly of short peptides might significantly impact their biochemical properties [5, 12]. Despite available data on safety for similar conjugates, the clinical implementation of new radiopharmaceuticals should start with a thorough evaluation of safety and dosimetry.

Here, we report the experience of the first-in-human clinical trial concerning the safety and biodistribution of a [^68^Ga]Ga-NOTA-PEG_2_-RM26-based tracer in prostate and breast cancer patients using PET/CT.

## Materials and methods

### Study participants

Twelve subjects, six men with newly discovered hormone-naive PC and six women with ER + metastatic BC, were recruited for the study. Inclusion criteria included biopsy-proven advanced-stage cancer, age > 18, Eastern Cooperative Oncology Group performance status of 0 or 1, adequate bone marrow, renal and hepatic function, and no clinical signs of heart failure. Exclusion criteria included pregnancy or breastfeeding, recent significant cardiovascular event or intervention, and participation in another clinical trial evaluating an investigational medical product within four weeks.

All study participants provided written informed consent. The study was approved by the Swedish Ethical Review Authority and the Swedish Medical Products Agency (Dnr: 2021-06886-01, EudraCT: 2021-004980-28) and registered on ClinicalTrials.gov (NCT06147362).

### Monitoring for safety

Pulse, blood pressure and body temperature were monitored before, during, and after the examination. Thresholds on pulse increase and blood pressure decrease of 10% were predefined to prompt investigation of possible anaphylaxis. After leaving the hospital, subjects were encouraged to contact the clinic or seek emergency medical attention if they experienced any possible reaction the first 24 h after the examination. All study participants were further interviewed 2 weeks after examination to identify any potential side effects.

### Radiotracer synthesis

Preparation and cGMP-compliant radiolabelling of [^68^Ga]Ga-NOTA-PEG_2_-RM26 was performed according to Jussing et al. [13]. The product fulfilled specifications and had a radiochemical purity of > 95% before being released for administration.

### PET/CT protocol

Subjects were intravenously administered an activity of 2 MBq/kg of [^68^Ga]Ga-NOTA-PEG_2_-RM26. PET acquisitions were performed at 5-, 20-, 40-, 60-, 120-, and 180 min post-injection. The first three scans were performed from the eyes to mid-thigh to achieve good time resolution, and the last three were whole-body scans. Bed position times were adjusted from 30 s/bed for the first scans up to 2.5 min/bed for the last scans to compensate for radioactive decay. CT scans (tube voltage 120 kV, noise index 32, pitch 0.984, rotation time 0.5 s) were performed for attenuation and localization purposes. Participants were asked to void hourly, and urine activity was measured using a gamma counter (Hidex, Turku, Finland).

PET scans were reconstructed using ordered subset expectation maximization (3 iterations, 16 subsets) and a 5.5 cm Gaussian postprocessing filter. Time of flight and point spread function were applied to reconstructions, as well as corrections for attenuation, scatter, randoms, normalization, and deadtime. Imaging was performed on a GE Discovery MI4 PET/CT system (GE Healthcare, Milwaukee, WI, USA).

### Biodistribution

The biodistribution of [^68^Ga]Ga-NOTA-PEG_2_-RM26 was determined for organs with higher uptake than muscle. Included organs were segmented to determine the total activity for each time point. The activity was decay corrected to the time of injection. For organs with homogenous activity distribution (liver and spleen), total activity was determined by multiplying the organ volume based on CT scans with the mean activity concentration in a representative spherical volume of interest (VOI). The total activity for the pancreas and gall bladder was determined with a threshold-based (10%) VOI to account for inhomogeneous activity distribution. Furthermore, threshold based VOIs were used to determine the parenchymal kidney activity. This was done by subtracting the renal pelvis from the total renal VOI. Total activity in blood was achieved from activity concentration in the thoracic aorta multiplied by sex and weight-adjusted reference blood volume. Sex and weight-adjusted reference blood volume was estimated using 75 ml/kg body weight for men and 65 ml/kg body weight for women [14]. Total renal excretion was based on the collected urine. The biological half-lives were calculated by fitting a mono- or biexponential curve to the mean values of both cohorts. Image analyses were conducted in the Hermes Affinity viewer (Hermes Medical Solutions, Stockholm, Sweden).

## Results

### Participants

The included subjects are summarised in Table 1. Participants with BC were determined metastatic at the time of recruitment. Participants with PC were hormone naive de novo cancers with high-risk or very high-risk cancers (grade 4–5, according to the International Society of Urological Pathology). The mean and standard deviation of the administered mass of [^68^Ga]Ga-NOTA-PEG_2_-RM26 was 23.5 ± 8.0 µg (range, 12–36 µg). The mean administered activity was 157 ± 27 MBq (range, 113–194 MBq).

**Table 1:** Demographics of study participants

**Breast cancer (** ***n*** ** = 6)**	Median (range)
Age	69 (43–82)
Weight (kg)	70.3 (53–86)
BMI	24.9 (21.2–28.7)
Injected activity/body weight (MBq/kg)	2.01 (1.83–2.14)
Distant metastases (bone, lymph nodes) Tumour biology: ER+, HER2-.	100%
Ongoing antitumoral treatment*	0%
**Prostate Cancer (** ***n*** ** = 6)**	**Median (range)**
Age	67 (57–80)
Weight (kg)	93 (71–113)
BMI	29.2 (20.0–33.0)
Injected activity/body weight (MBq/kg)	1.93 (1.68–2.23)
De novo prostate cancer	100%
PSA (µg/l)	12.5 (6.1–125)
Gleason Score	8.5 (4 + 3 to 5 + 5)
ISUP Score	4.5 (3–5)
Stage	T2-4, N1, M1 except for one patient that was 0

Abbreviations: ER + = estrogen receptor positive, HER2- = human epidermal growth factor receptor 2 negative. * Systemic therapy; chemo- and/or antihormonal therapy

### Safety

Subjects tolerated injections well. Table 2 shows the subjects’ body temperature, heart rate, and systolic blood pressure during the examination. Body temperatures remained stable. One participant (BC1) showed an increase at the endpoint of the examination, both concerning heart rate (22%) and systolic pressure (41%). Another participant (PC4) showed a decrease in systolic blood pressure (-18%) during the examination, but the diastolic pressure remained stable throughout, ruling out anaphylaxis. No adverse events were observed during the PET examination. One of the breast cancer patients experienced an aggravation of already pre-existing chest pain the evening after the study PET/CT examination. She was referred to the Emergency Department where an acute CT was done, that ruled out acute cardiopulmonary pathology or complications related to the investigation. Skeletal metastases were concluded to be the reason for the pain. The event was considered as an adverse event unrelated to the investigational procedure or the investigational medical product. No other participant reported any adverse event related to the study during the two-week telephone interview.

**Table 2:** Body temperature, heart rate, and blood pressure in participants during PET/CT examination

	Body temperature, °C			Pulse beats per minute			Blood pressure, mmHg		
Study participant	Initial	Mid-exam	End of exam	Initial	Mid-exam	End of exam	Initial	Mid-exam	End of exam
PC1	36.8	36.3	36.9	92	75	76	150/91	164/105	157/93
PC2	37.3	37.5	37.6	90	95	96	133/78	131/77	145/87
PC3	36.3	36.6	36.9	58	56	52	126/78	118/70	145/88
PC4	36.4	36.1	36.7	65	60	59	140/79	115/73	141/84
PC5	37.0	36.5	37.2	58	63	60	158/88	179/84	167/82
PC6	36.8	36.8	36.7	69	55	50	164/94	147/90	151/98
BC1	36.4	36.0	36.4	86	91	105	123/72	156/83	173/81
BC2	35.8	36.0	37.0	70	57	72	122/81	120/83	131/87
BC3	37.0	35.7	36.6	72	67	66	134/77	143/82	141/72
BC4	36.5	36.1	35.9	93	94	80	137/76	168/77	154/83
BC5	36.5	37.2	37.1	76	63	66	147/89	134/79	141/84
BC6	37.1	36.6	36.6	74	69	72	135/101	128/92	128/90

### Biodistribution

**Fig. 1:**
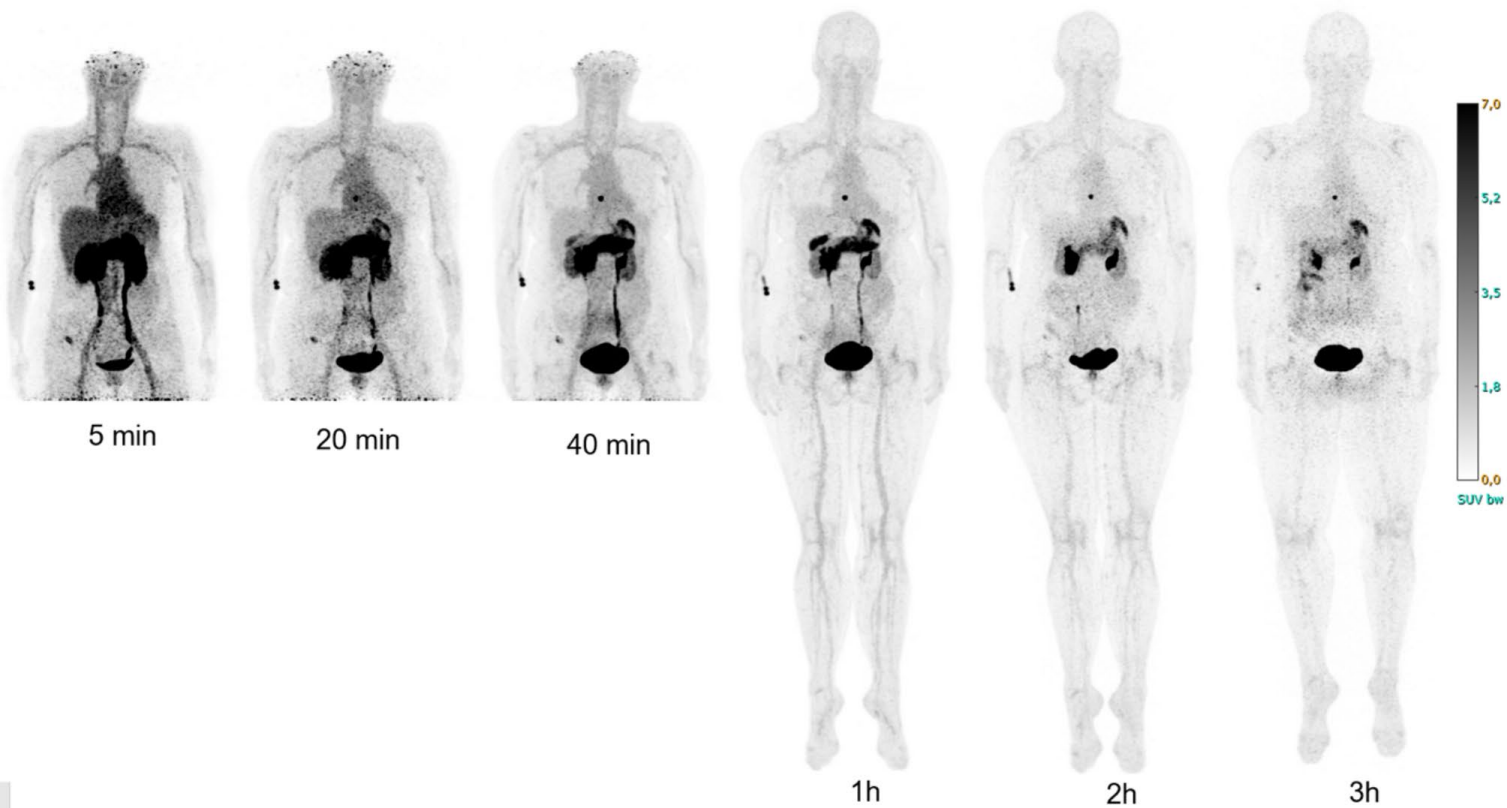
Maximum intensity projection images of a female participant with metastatic estrogen-positive breast cancer at different time-points post injection

**Fig. 2:**
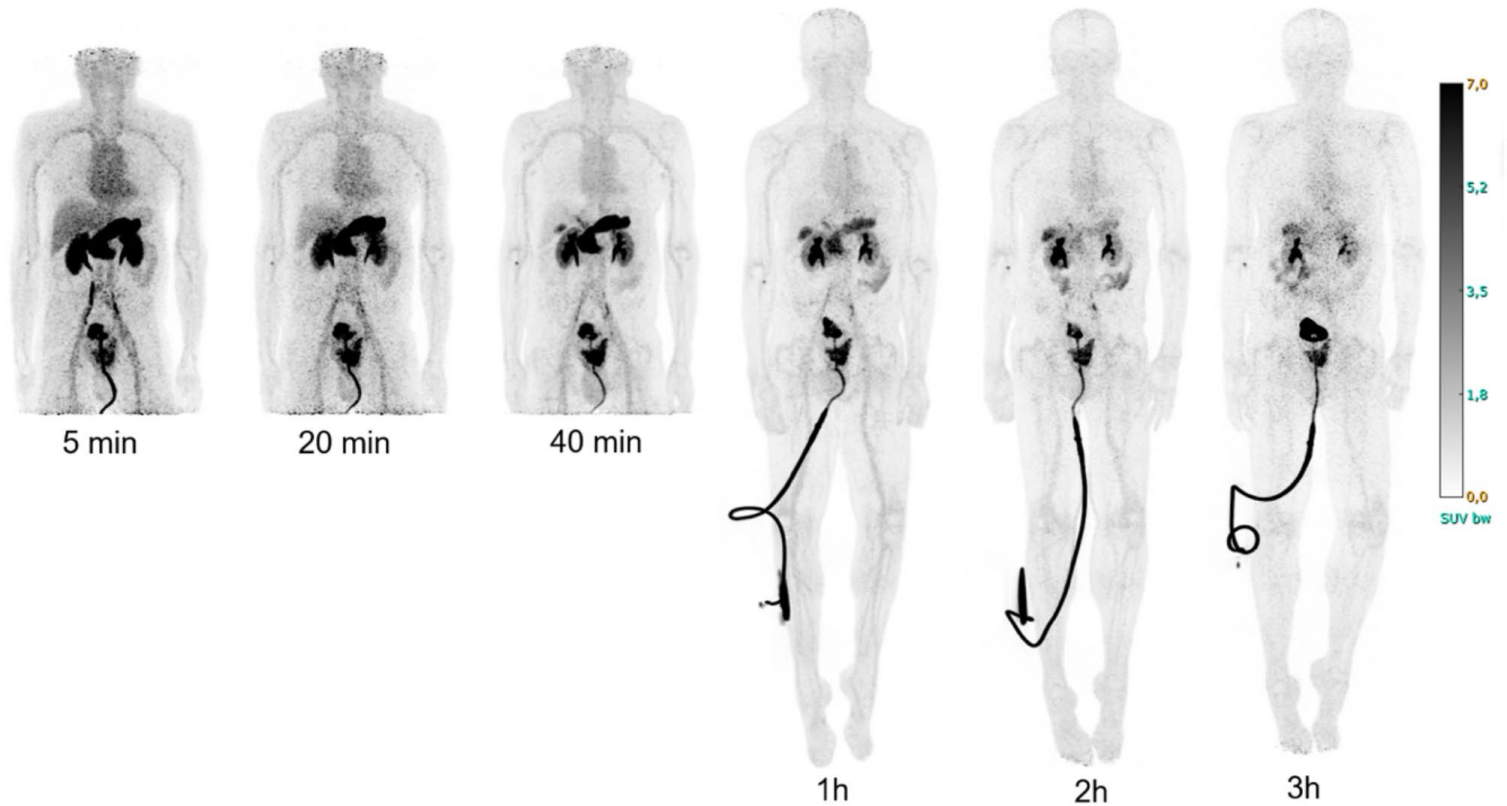
Maximum intensity projection images of a male participant with high-risk, hormone naive recently diagnosed prostate cancer at different time-points post injection. The current patient had a urinary catheter during the examination

Typical biodistributions of [^68^Ga]Ga-NOTA-PEG_2_-RM26 in participants from both cohorts are shown in Figs. 1 and 2. Initial uptake is high in the pancreas and kidneys and moderate in the liver, heart, spleen, and rectum. Small bowel and lungs show low uptake. Activity concentration in the gall bladder increases with time after injection.

**Fig. 3:**
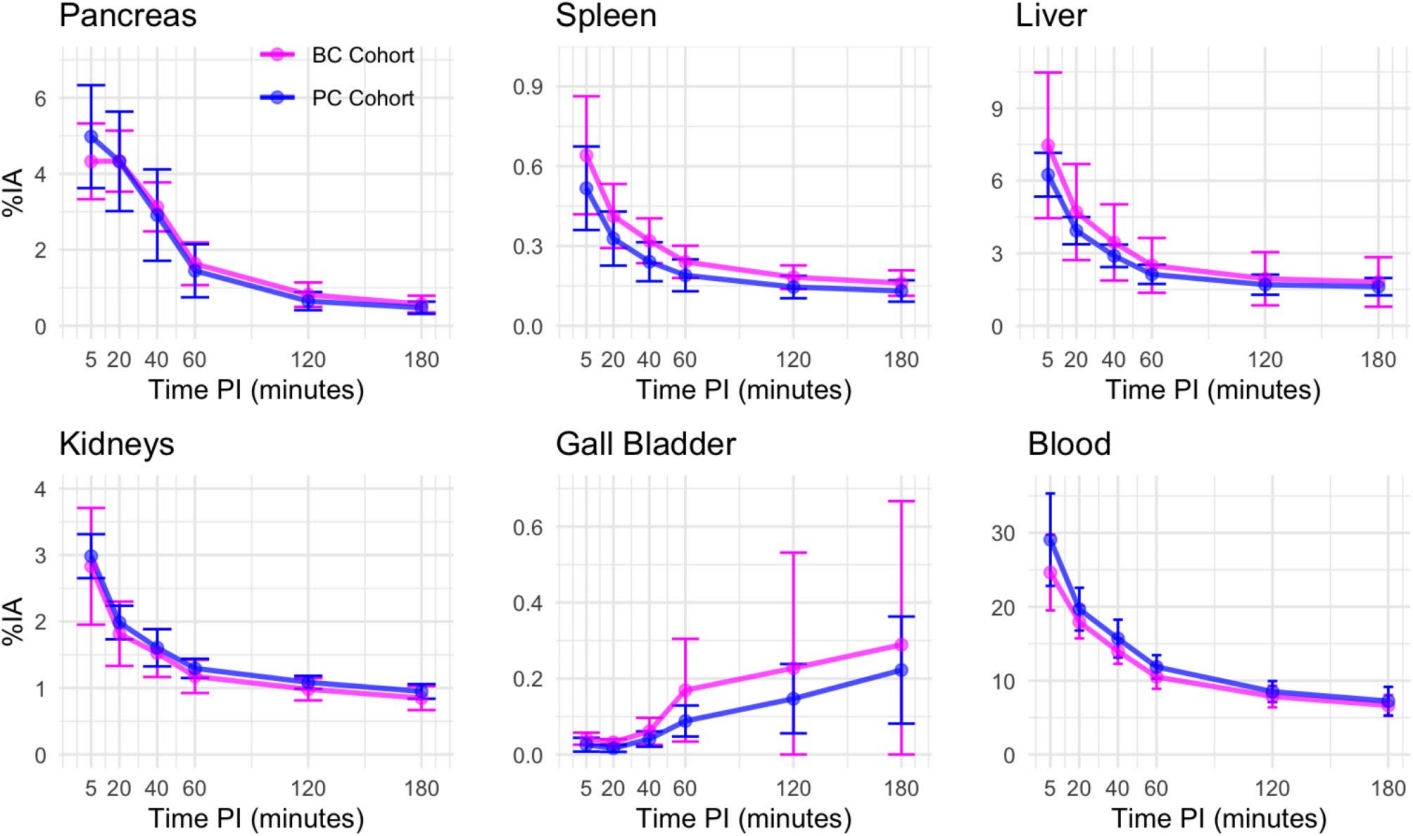
Percent of injected activity (%IA) as a function of time after injection for the pancreas, spleen, liver, kidneys, gall bladder, and blood pool. Pink markers show the mean for the breast cancer cohort and blue for the prostate cancer cohort. The error bars show the standard deviations. PI: post injection

The uptake and excretion in the pancreas, liver, spleen, renal cortex, gall bladder, and blood pool are shown in Fig. 3. A maximum of 5% of the injected activity is seen in the pancreas followed by a mono exponential excretion with biological half-life of 43 min.

Liver, spleen, kidney cortex, and blood showed a biexponential behavior of the excretion. In the liver a maximum 7% of the injected activity was seen followed by excretion with biological half-lives of 14 min (72%, rapid phase) and 6 h (28%, slow phase). The renal cortex accounted for up to 3% of the injected activity and the biological half-lives were 10 min (59%, rapid phase) and 3.5 h (41%, slow phase). The corresponding values for the spleen were 12 min (65%, rapid phase) and 3.5 h (35%, slow phase) and for blood, 15 min (59%, rapid phase) and 3.1 h (41%, slow phase). For the BC and PC cohorts, total excreted activity via urine after three hours was 59% ±12% and 59% ±11% of injected activity, respectively.

## Discussion

Our study shows that injecting [^68^Ga]Ga-NOTA-PEG_2_-RM26 into participants with BC and PC was safe. This is in keeping with observations by Chernov et al. [10] using [^99m^Tc]Tc-maSSS-PEG_2_-RM26 and Zhang et al. [9] using [^68^Ga]Ga-NOTA-PEG_3_-RM26.

Uptake in the pancreas was high compared to other normal organs. This was expected since the pancreas has high natural expression of GRPR [15]. The main route of excretion was via urine and, to a lesser extent, the hepatobiliary system which reflects the common behaviour of many relatively small molecules. Of notice was the rapid tracer uptake in pancreas and kidneys followed by urinary excretion already on the 5-minute scan. Six subjects had high urinary excretion to such a degree that a halo artefact around the bladder could be seen. Zhang et al. also briefly discuss this and suggest administering furosemide. This seems reasonable, especially in prostate cancer patients who have a high risk of pelvic metastases.

This first-in-human study included only twelve patients, and very few visible uptakes were suspected to be metastatic. Uptake in these metastases is unlikely to affect biodistribution in normal organs. However, biopsy verification of GRPR expression in visible lesions is necessary to evaluate the diagnostic performance of a new radiopharmaceutical.

Chernov et al. reported differences in whole-body elimination due to quicker passage through the gastrointestinal (GI) canal in males but equally eliminated via blood between sexes. The current study did not show any differences between the two cohorts concerning excreted activity in urine, distribution in blood or the passage of activity in the GI canal.

[^68^Ga]Ga-NOTA-PEG_2_-RM26 demonstrates low to moderate uptake in the liver, bowel, bones, and lungs which could be significant for identifying primary cancers or metastatic lesions in these regions. Therefore, this agent could serve as a valuable complement to existing diagnostic methods as well as to be further explored as a companion diagnostic for the theranostic purposes. As an example, the different uptake of GRPR-targeting radiopharmaceuticals in comparison to PSMA-targeting radiopharmaceuticals, may, in the future, offer new therapeutic options that complement the current [^177^Lu]Lu-PSMA treatments.

## Conclusion

[^68^Ga]Ga-NOTA-PEG_2_-RM26 is safe for participants with BC and PC, paving the way for further clinical investigations in molecular oncologic imaging.

## Data Availability

The datasets generated and analysed during the current study are available from the corresponding author on reasonable request.

## Declarations

### Clinical trial registration

ClinicalTrials.gov NCT06147362. Registered 17 Nov 2023 retrospectively registered.

## Abbreviations

BC: Breast cancer
CT: Computed tomography
ER+: Estrogen receptor positive
GI: Gastrointestinal
GRPR: Gastrin releasing peptide receptor
HER2-: Human epidermal growth factor receptor 2 negative
IA: Injected activity
PC: Prostate cancer
PET: Positron emission tomography
PI: Post injection
VOI: Volume of interest
